# Railway Actuator Made of Magnetic Elastomers and Driven by a Magnetic Field

**DOI:** 10.3390/polym10121351

**Published:** 2018-12-06

**Authors:** Yasuhiro Umehara, Yusuke Yamanaga, Shota Akama, Shunsuke Kato, Shogo Kamoshita, Mika Kawai, Tetsu Mitsumata

**Affiliations:** 1 Graduate School of Science and Technology, Niigata University, Niigata 950-2181, Japan; umehara.yasuhiro.22@rtri.or.jp (Y.U.); aaa.rock@icloud.com (S.A.); k-a-shunsuke.6203@i.softbank.jp (S.K.); mikagoro@eng.niigata-u.ac.jp (M.K.); 2 Running Gear, Vehicle Structure Technology Division, Railway Technical Research Institute, Tokyo 185-8540, Japan; yamanaga.yusuke.14@rtri.or.jp; 3 ALCA, Japan Science and Technology Agency, Tokyo 102-0076, Japan; 4 Vehicle Noise and Vibration, Vehicle Structure Technology Division, Railway Technical Research Institute, Tokyo 185-8540, Japan; kamoshita.shogo.70@rtri.or.jp

**Keywords:** soft material, stimuli-responsive gel, magnetic elastomer, railway vehicle

## Abstract

We fabricated a mono-link using bimodal magnetic elastomers that demonstrate drastic changes in the elastic modulus by magnetic fields. The magnetic elastomer is bimodal consisting of large magnetic particles and nonmagnetic fine particles. The storage modulus for bimodal magnetic elastomers was altered from 2.2 × 10^5^ to 1.7 × 10^6^ Pa by a magnetic field of 500 mT. Compression tests up to a strain of 20% also revealed that the on-field stress for the bimodal magnetic elastomer was 1.24 times higher than the off-field stress. The bimodal magnetic elastomer was synthesized for the mono-link and was mounted on the bogie of a railway vehicle. A running test exhibited that the wheel lateral force was reduced by 20% by applying a magnetic field of 390 mT.

## 1. Introduction

Soft materials responsive to external stimuli, such as temperature, pH, and electric fields have attracted considerable attention as next-generation actuators. The soft material we report here is called magnetic elastomer and it demonstrates dramatic changes in viscoelastic properties in response to a magnetic field, which is called the magnetorheological (MR) effect [1,2,3,4,5,6,7,8,9,10,11,12,13]. The magnetic elastomer consists of polymeric matrices with a low elastic modulus and magnetic particles with high magnetization. When a magnetic field is applied to the magnetic elastomer, the magnetic particles align in the direction of the magnetic field and make chain structures within the cross-linked matrix. The apparent elastic modulus of magnetic elastomers increases by a magnetic field due to both the magnetic interaction and stress transfer among the magnetic particles.

Magnetic particles under magnetic fields are connected by weak magnetic interaction and therefore the chain of magnetic particles is mechanically fragile. At the linear viscoelastic regime, normally under strains below 0.1%, the particle forms a chain-like structure. However, most of the chains are destroyed by a strain of 100% [14], which is called the magnetic field-enhanced Payne effect [15,16,17] seen in polymer composites containing a large amount of fillers. This deterioration of the MR effect seen under high strains has thus far been restricted in terms of its practical use as an industrial material. We have shown that this defect can be improved by using bimodal magnetic elastomers in which magnetic and nonmagnetic particles are dispersed [18,19,20]. We reported a bimodal magnetic elastomer containing 9.6 vol % of nonmagnetic ZnO particles that demonstrates significant increase (24.6 to 152 kPa) in the stress even under a high strain of 20% at 420 mT [18]. This strongly indicates that the nonmagnetic particles reinforce the fragile structure of chains of magnetic particles. Similar reinforcement by nonmagnetic particles was also observed in the dynamic viscoelastic measurement of gels [19]. This advantage seen in bimodal magnetic elastomers can be used for industrial applications when a large force is applied to the elastomer, e.g., products for railway vehicle and railroads.

The purpose of this study was to develop a material showing a large increase in the elastic modulus by magnetic fields even though the material has high elastic modulus at no magnetic fields. Generally, a hard magnetic elastomer is not changed significantly by magnetic fields because magnetic particles cannot make chain-like structures within the hard matrix. Another purpose of this study was to demonstrate the fact that magnetic elastomer with a high elastic modulus can be employed in actuators for railways, where huge loads are applied. In the present study, we demonstrated our first application of bimodal magnetic elastomers to a mono-link for railway vehicles where large forces as high as 10 kN are applied. The mono-link was mounted between the bogie frame and the axle box of the railway vehicle so as to enable the steering of the bogie. Generally, the material used in the present mono-link of railway vehicles is styrene-butadiene rubber with a constant elastic modulus. Since the elastic modulus of the rubber is very high, the wheel can rotate only within a small angle due to the deformation of the rubber. If the rubber is very soft, the wheel can rotate at wide angles, resulting in less friction between the wheels and rails. However, the softness simultaneously causes travelling instability when the vehicle travels in straight line at high speed. If the elastic modulus of the materials embedded in the mono-link can be changed freely, the vehicle will have less friction in curved sections and high stability when travelling in a straight line by optimizing the elasticity of the material. The results obtained from our numerical simulation [21] revealed that the material of this actuator needs an off-field modulus higher than few 100 kPa and high relative changes in the modulus 2–5-fold higher than the off-field modulus. This material can reduce the lateral force acting on the outer rail by 20% in a curved section with a radius of curve of 200 m. In this study, we did not prepare a magnetic circuit that is a special for railway actuators. A magnetic field of 389 mT was applied to magnetic elastomer using two pieces of permanent magnet for one mono-link. We carried out a running test using a railway vehicle equipped with the magnetic elastomer at a test line. The effect of the magnetic fields on the viscoelastic and mechanical properties for bimodal magnetic elastomers containing nonmagnetic particles is shown, and the running performance of the test vehicle is presented.

## 2. Experimental Procedures

### 2.1. Synthesis of Magnetic Elastomers

**Figure 1:**
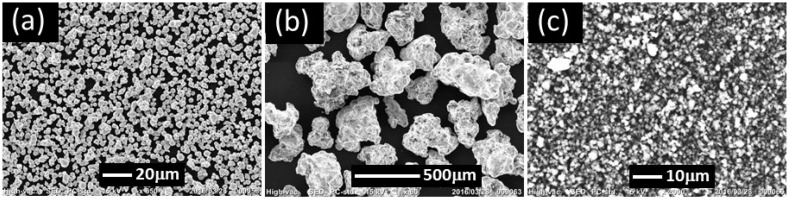
SEM photographs of magnetic and nonmagnetic particles. (**a**) SP, (**b**) LP, and (**c**) NP.

Monomodal and bimodal magnetic elastomers were synthesized using a prepolymer method. Polypropylene glycols (*M*_w_ = 2000, 3000), prepolymer cross-linked by tolyrene diisocyanate (Wako Pure Chemical Industries, Ltd., Osaka, Japan), a plasticizer (dioctyl phthalate, DOP, Wako Pure Chemical Industries. Ltd., Osaka, Japan), magnetic particles, and nonmagnetic particles were mixed by a mechanical mixer for several minutes. Carbonyl iron particles with a diameter of 7.0 μm (CI, CS grade, BASF SE., Ludwigshafen, Germany) or iron particles with a diameter of 235 μm (Somaloy 700, Höganäs Japan K.K, Fukaya, Japan) were used for magnetic particles. Aluminum hydroxide Al(OH)_3_ was used for nonmagnetic particles (Wako Pure Chemical Industries. Ltd., Osaka, Japan). The abbreviations for the small magnetic particles, large magnetic particles, and nonmagnetic particles are SP, LP, and NP, respectively. The weight concentration of the magnetic particles (SP or LP) and NP was kept at 80 and 8.0 wt.%, respectively, which corresponds to a volume fraction of 0.38 and 0.13, respectively. The weight concentration of the plasticizer to the polyurethane matrix was kept at 30 wt.%. The mixed solution was poured into an aluminum mold and cured in an oven at 120 °C for 3 h. The median diameter of these particles was measured using a particle size analyzer (SALD-2200, Shimadzu, Kyoto, Japan). The densities of SP, LP, and NP measured were 7.57, 8.6, and 2.4 g/cm^3^, respectively. Scanning electron microscope (SEM) observation was carried out using JCM-6000 Neoscope (JEOL, Akishima, Japan) with an accelerating voltage of 10 kV without Au coating. SEM images of magnetic (SP and LP) and nonmagnetic (NP) particles are shown in Figure 1. We investigated the effect of particle size on the MR effect for magnetic elastomers and found that the MR effect for magnetic elastomers with large particles was significant compared to small particles [13]. For example, the relative change in storage modulus was 194-fold for magnetic elastomers with magnetic particles of 235 μm. On the other hand, the relative change in storage modulus was 58-fold for magnetic elastomers with magnetic particles of 2.5 μm. It is a feature of large magnetic particles that the linear viscoelastic regime is extended to high strains compared to small magnetic particles. In addition, the change in loss factor for magnetic elastomers with magnetic particles of 235 μm was much higher than that for magnetic particles of 2.5 μm. Due to these advantages of large magnetic particles, we decided to employ large magnetic particles for the material of the railway actuator.

### 2.2. Dynamic Viscoelastic Measurement and Compression Test

Dynamic viscoelastic measurement was carried out for the magnetic elastomers using a rheometer (MCR301, Anton Paar, Graz, Austria) at 20 °C. The strain was constant at 0.01% and the frequency was kept at 1 Hz. The sample was a disk 20 mm in diameter and 1.5 mm in thickness. The normal force initially applied to the magnetic elastomer was approximately 0.4 N. The stress–strain experiment was performed for magnetic elastomers at room temperature using a compression apparatus (EZ-SX, Shimadzu, Kyoto, Japan). The measurement was carried out at strains below 22% with a compression speed of 100 mm/min. The sample was a cylinder 15 mm in diameter and 5 mm in thickness. A magnetic field of 480 mT was applied using a permanent magnet 15 mm in diameter and 10 mm in thickness (NeoMag Co. Ltd., Ichikawa, Japan), which was set on the bottom of the magnetic elastomer via a thin plastic disk with 0.40 mm thickness.

## 3. Results and Discussion

### 3.1. Magnetic Field Response in Shear Modulus

**Figure 2:**
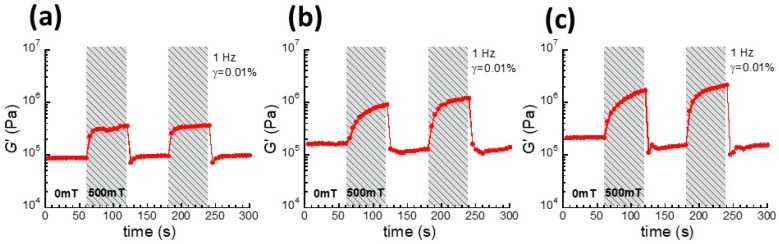
Magnetic field responses of the storage modulus under the linear viscoelastic regime for magnetic elastomers containing (**a**) SP, (**b**) LP, and (**c**) LP+NP.

Figure 2a–c shows the magnetic-field response of the storage modulus of magnetic elastomers with SP, LP, and LP+NP at a low strain of 0.01%. A magnetic field of 500 mT was applied to the magnetic elastomers every 60 s. It was observed for all magnetic elastomers that the storage modulus changed synchronously with the magnetic field. In Figure 2a, the magnetic elastomer containing SP demonstrated a small MR effect ranging from 8.8 × 10^4^ to 3.6 × 10^5^ Pa. The relative change in the storage modulus *G*′_500_/*G*′_0_ was 4.1, which is a typical value for monomodal magnetic elastomers containing carbonyl iron particles with several microns with an off-field modulus of ~10^4^ Pa. In Figure 2b, the magnetic elastomer with LP demonstrated a significant MR effect ranging from 1.7 × 10^5^ to 9.3 × 10^5^ Pa. This is consistent with our previous report in that magnetic elastomers containing iron particles with a large diameter (235 μm) exhibited a significant MR effect compared to magnetic particles of several microns [13]. In Figure 2c, the bimodal magnetic elastomer with LP and NP demonstrated a giant MR effect ranging from 2.2 × 10^5^ to 1.7 × 10^6^ Pa, which also coincides with our previous report that the addition of nonmagnetic particles enhances the MR effect for elastomers [18,20] and hydrogels [19]. It was considered that the discontinuous chains of magnetic particles are connected via nonmagnetic particles. Al(OH)_3_ particles are randomly dispersed in the polyurethane matrix, while ZnO particles form agglomerates in the matrix. Under magnetic fields, the storage modulus for magnetic elastomers with Al(OH)_3_ particles was higher than that for ZnO particles, suggesting that Al(OH)_3_ particles make chains more easily than ZnO particles. For applications on railways, a high elastic modulus under magnetic fields is needed to endure under high loads. Al(OH)_3_ also has an advantage of production cost because it can be recycled from the sludge of lightweight metals. The relative changes in the storage modulus, *G*′_500_/*G*′_0_ for magnetic elastomers with LP/NP particles was 7.7. It is not usual that such a large increase in storage modulus is observed for magnetic elastomers with an off-field modulus that is higher than 10^5^ Pa. We reported that magnetic hydrogels with a polysaccharide matrix exhibited a similar large response against magnetic fields [22]. The storage modulus after the first application of the magnetic field decreased slightly from the original modulus, indicating that the particle network was slightly destroyed by the magnetic field. However, the movement of magnetic particles occurred within the limitations of elastic deformation without causing the destruction of the polymer network. It was also found that magnetic elastomers with LP and LP+NP demonstrated high energy dissipation. The mechanical loss factor at 0 mT in the linear viscoelastic regime for SP, LP, and LP+NP was 0.11, 0.10, and 0.17, respectively. At 500 mT, the loss factor for SP, LP, and LP+NP was 0.15, 0.49, and 0.43, respectively.

### 3.2. Magnetic Field Response in Compressional Modulus

**Figure 3:**
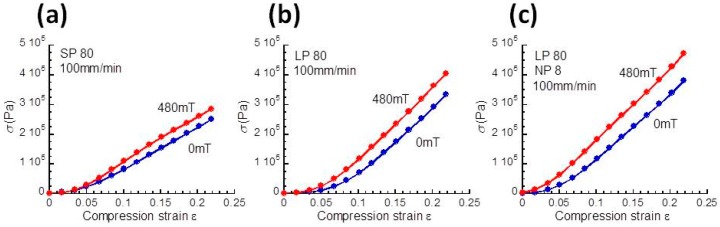
Strain–stress curves at 0 and 480 mT for magnetic elastomers containing (**a**) SP, (**b**) LP, and (**c**) LP+NP.

Figure 3a–c shows the stress–strain curves for magnetic elastomers with SP, LP, and LP+NP in the absence and in the presence of a magnetic field of 480 mT. All magnetic elastomers exhibited typical MR behavior in that the on-field stress was higher than the off-field stress in the whole range of strains. The Young’s modulus for the magnetic elastomers was determined by the slope of the strain–stress curves under strains below 7%. Table 1 shows the effect of magnetic field on the Young’s modulus and stress under a strain of 22% for magnetic elastomers containing SP, LP, and LP+NP. The Young’s modulus at 480 mT for LP+NP was approximately twice that of the others, even though it showed the highest Young’s modulus at 0 mT. It was also found for LP+NP that the stress at a strain of 0.22 or the relative change in the stress *σ*_480_/*σ*_0_ was the highest despite its high off-field stress. These results basically coincide with the result of the storage modulus obtained from the dynamic viscoelastic measurements. However, the relative change in Young’s modulus or the stress was clearly lower than that calculated from the storage modulus as shown in Figure 2. The inconsistency in the MR effect on the storage modulus and Young’s modulus was not clearly due to the demagnetizing field. In the measurement of the Young’s modulus, the magnetic field was applied from the bottom of the magnetic elastomer by a permanent magnet. Therefore, the strength of the magnetic field decreased remarkably with the thickness to the top of elastomer. Conversely, in the measurement of the storage modulus, the gap between the magnetic poles was a few millimeters, therefore, the magnetic field was rather uniform. We considered the attenuation of the field strength to be a main reason that the MR effect determined from the Young’s modulus was far lower than that from the storage modulus. For the application to railway vehicles, the important parameters were both the stress at 0 mT and the change in stress at high strains, because a large stress was applied to the elastomers. We decided to use magnetic elastomers of LP+NP as a material for the bush to be mounted on the bogie of railway vehicles. This experiment using railway vehicles is not permitted in cases where there is a possibility of derailment accidents.

### 3.3. Fabrication of Actuators for Railways

**Figure 4:**
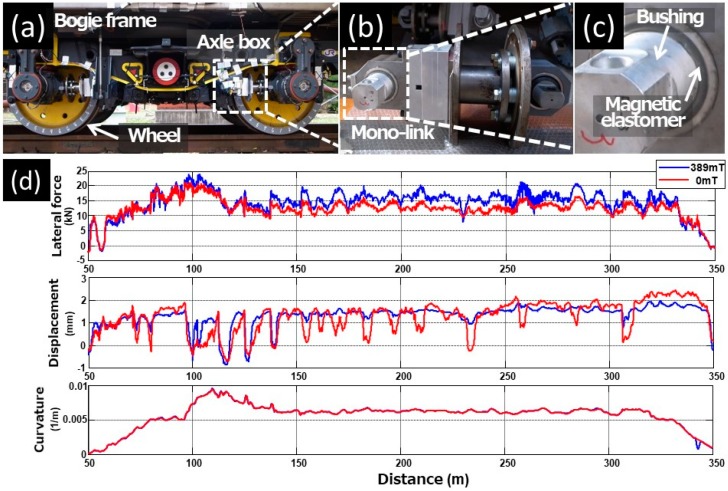
Photographs of (**a**) bogie and (**b**,**c**) mono-link made of magnetic elastomer containing LP+NP, (**d**) lateral force (**top**), displacement of the magnetic elastomer bushing (**middle**), and curvature (**bottom**) as a function of running distance.

Figure 4a demonstrates a photograph representing a test bogie with the mono-link of magnetic elastomers described in the previous section. The mono-links were installed in four places between the bogie frame and the axle box. The magnetic elastomer is a bimodal elastomer consisting of LP and NP and was molded in the bushing for the mono-link, as shown in Figure 4b,c. In a running test, a diesel locomotive hauled the test car with the test bogie on the front side and the normal bogie on the rear side. The running test was carried out using the test line with a test curve section (radius: 160 m, cant: 90 mm) at a velocity of approximately 15 km/h. 

Figure 4d exhibits the effect of the magnetic field on the outer wheel lateral force of the leading wheelset, the outer magnetic elastomer bushing’s displacement of the leading wheelset, and the curvature by the distance on the horizontal axis. Lateral force is one of the forces acting between the wheel and the rail, and, in general, the smaller the lateral force, the better the curve passing performance of a vehicle. It was observed that the outer wheel lateral force at 0 mT was reduced in the circular curve section (distance: 150 ~ 300 m) by approximately 20% compared to that at 389 mT. From the previous simulation results [21], it is inferred that the mobility of the wheelsets changed due to the change in the elastic modulus depending on the presence or absence of the magnetic field, although it is possible that the causes of the change in lateral force may include the influence of the friction coefficient produced by the temperature or the humidity on the test day. Moreover, from the result of the displacement of the magnetic elastomer bushing at 0 mT, it is evident that it is easily affected by track irregularity due to the reduction of the longitudinal stiffness of the mono-link. 

## 4. Conclusions

We fabricated bimodal magnetic elastomers containing both large magnetic particles and fine nonmagnetic particles for railway vehicles and investigated the magnetic-field effect on the elastic properties. The bimodal magnetic elastomers demonstrated a significant response to the magnetic fields both for the storage modulus and stress under 20% strain, showing that the chains of the magnetic particles were bridged by nonmagnetic particles. We succeeded in fabricating mono-links for railway vehicles consisting of the bimodal magnetic elastomer and carried out a running test on a test line. The test vehicle successfully ran at a speed of 15 km/h in a curved section with a curvature of 160 m. It was revealed that the lateral force applied to the rail was reduced by 20% by applying a magnetic field. We stress here that magnetic elastomers can actually be used as a stimuli-responsive material for industry.

## Figures and Tables

**Table 1:** Effect of the magnetic field on the Young’s modulus and stress at a strain of 0.22 for magnetic elastomers containing SP, LP, and LP+NP.

Particles	Young’s Modulus *E* (kPa)			Stress at 0.22 *σ* (kPa)		
	0 (mT)	480 (mT)	*E*_480_/*E*_0_	0 (mT)	480 (mT)	*σ*_480_/*σ*_0_
SP	567.7	784.2	1.38	250.0	285.5	1.14
LP	339.0	712.0	2.10	334.3	404.3	1.21
LP+NP	752.7	1461	1.94	381.2	472.8	1.24
